# Supplementary material for: A Global Screen for Assembly State Changes of the Mitotic Proteome by SEC-SWATH-MS
Source: Cell Syst. 2020 Feb 26;10(2):133–155.e6. doi: 10.1016/j.cels.2020.01.001 (PMC7042714; doi:10.1016/j.cels.2020.01.001)

P29590 | PML_HUMAN | PML MYL PP8675 RNF71 TRIM19
Monomer MW [kDa]: 97.551 Monomer expected elution fraction: 40

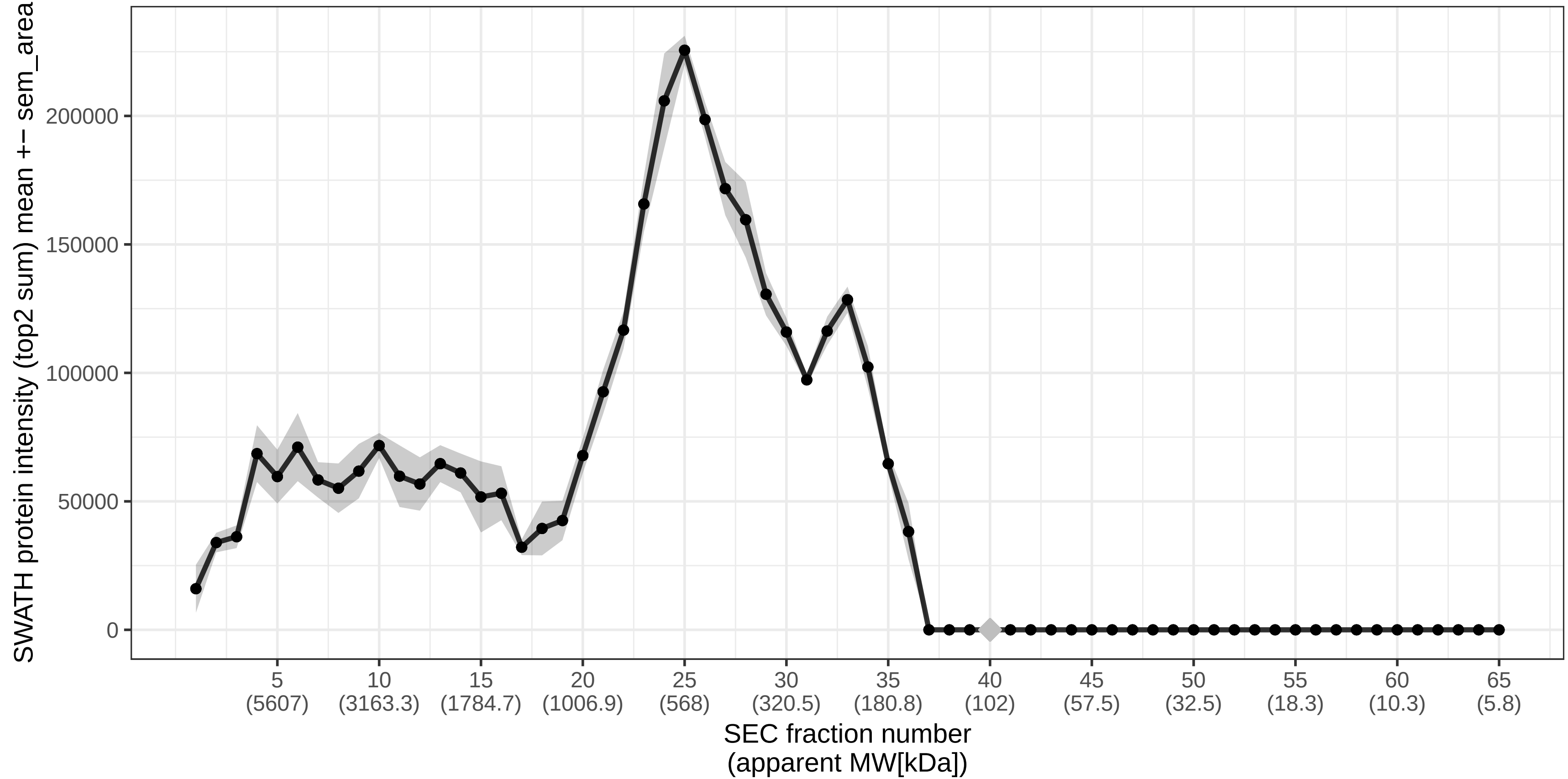

Supplement: Data S1. SEC-SWATH-MS Protein Chromatograms, Related to Figure 1 [file mmc6.zip › SECchrom_P29590_PML_HUMAN_PML_MYL_PP8675_RNF71_TRIM19.pdf]
